# Case Report: Methylprednisolone-induced pheochromocytoma crisis resulting in cardiac arrest

**DOI:** 10.3389/fmed.2026.1779740

**Published:** 2026-06-03

**Authors:** Zhenzhen Hu, Lianglei Hou, Anwu Huang

**Affiliations:** 1 Department of Intensive Care Unit, Wenzhou Central Hospital, Wenzhou, Zhejiang, China; 2 Department of Cardiology, Wenzhou Central Hospital, Wenzhou, Zhejiang, China

**Keywords:** cardiac arrest, case report, extracorporeal membrane oxygenation, methylprednisolone, pheochromocytoma crisis

## Abstract

Pheochromocytoma crisis, a rare yet life-threatening endocrine emergency, is characterized by acute hemodynamic instability that can lead to severe cardiovascular collapse, including cardiac arrest. Systemic glucocorticoid administration has been shown to trigger such crises in patients with pheochromocytoma. This report describes a 38-year-old female who developed symptoms including chest tightness, dyspnea, and vomiting shortly after receiving a methylprednisolone injection for urticaria, suggesting a possible association between glucocorticoid administration and the subsequent crisis. On admission, she suffered cardiac arrest and was managed with extracorporeal membrane oxygenation (ECMO). Coronary angiography, blood microbial RNA analysis, and metagenomic testing revealed no abnormalities. Despite comprehensive pharmacological treatment, the patient’s symptoms persisted, with recurrent ventricular fibrillation detected on electrocardiogram, prompting further investigations. A computed tomography (CT) scan identified an adrenal mass, and biochemical tests confirmed the diagnosis of pheochromocytoma. Following successful laparoscopic adrenalectomy, the patient experienced significant clinical improvement. This case demonstrates the potential for methylprednisolone to trigger or contribute to a pheochromocytoma crisis. However, it is important to acknowledge that other concurrent factors, such as use of medication, systemic inflammatory response, and physiological stress of resuscitative interventions, may have also played a role. The challenges posed by the diagnosis of this condition underscore the need for caution when administering glucocorticoids to patients with suspected pheochromocytoma.

## 1 Introduction

Pheochromocytoma is a neuroendocrine tumor originating from the chromaffin cells in the adrenal medulla. Its clinical relevance stems from the tumor’s ability to synthesize and release excessive amounts of catecholamines, which can lead to a range of cardiovascular and metabolic disturbances (1). Approximately 80%–85% of pheochromocytomas are found in the adrenal gland, with the remaining cases presenting as paragangliomas along the sympathetic nerve chain (2). Although rare, accounting for 0.2%–0.6% of hypertension cases (3), pheochromocytomas manifest with diverse and potentially life-threatening symptoms. Common signs, resulting from episodic or persistent catecholamine secretion, include severe headache, palpitations, and profuse sweating, collectively known as the “triple sign” (4). In some cases, patients may present solely with sustained hypertension or metabolic abnormalities (5).

In patients with pheochromocytoma, exposure to specific triggers can lead to a sudden and significant release of catecholamines, resulting in a life-threatening endocrine emergency known as pheochromocytoma crisis. This crisis is characterized by acute hemodynamic instability, manifesting as severe hypertensive episodes, fluctuating blood pressure, cardiogenic shock, acute pulmonary edema, arrhythmias, myocardial ischemia, and multi-organ dysfunction (6). Without timely recognition and intervention, the mortality rate is exceedingly high (7).

Notably, various exogenous factors can provoke a pheochromocytoma crisis, with drug-induced stimulation being a significant iatrogenic trigger. Previous studies have shown that the administration of systemic glucocorticoids in patients with pheochromocytoma can precipitate such a crisis (8). This case report describes a 38-year-old female who experienced a pheochromocytoma crisis and cardiac arrest after methylprednisolone injection for urticaria. While the temporal relationship suggests a possible triggering role of the glucocorticoid, it is important to consider other potential contributing factors, including the use of medication, systemic inflammatory response, and physiological stress of resuscitative interventions. The goal is to emphasize the need for caution when managing patients with known or suspected pheochromocytoma and to carefully weigh the risks and benefits of administering systemic glucocorticoids in these cases.

## 2 Case presentation

A 38-year-old female, previously in good health with no history of hypertension, diabetes, or other chronic conditions, presented to our hospital with a 3-day history of generalized rash and 8 h of difficulty breathing. Her height was 156 cm (weight: 47.5 kg). Three days before admission, she developed a generalized rash with no identifiable trigger. After taking oral ebastine 10 mg once daily at a local clinic, the symptoms improved. However, 2 days prior to admission, the rash recurred and became confluent. She was diagnosed with acute urticaria at a local health center and treated with a single intravenous dose of 80 mg methylprednisolone along with oral ebastine 10 mg. The urticarial symptoms partially improved, but 8 h before admission (at 15:00), she suddenly experienced chest tightness, dyspnea, and cold sweats. She was immediately taken to a local hospital, where her blood pressure was 147/106 mmHg, oxygen saturation was 60%, and heart rate was 160 bpm. She also vomited pink, frothy sputum. Emergency endotracheal intubation was performed, and she was transferred to our emergency department.

Upon arrival at our emergency department, the patient’s vital signs remained unstable. Her body temperature was 36°C, heart rate 135 bpm, blood pressure 90/55 mmHg, and oxygen saturation 45%. A series of tests were performed, with key laboratory results summarized in Table 1. The ECG revealed ventricular tachycardia (Figure 1), and bedside echocardiography showed nearly absent cardiac systolic function, with enlargement of the left atrium and ventricle. Given the possibility of acute myocardial infarction or fulminant myocarditis, emergency treatment was promptly initiated. Treatment measures included intravenous furosemide (20 mg) for diuresis, intravenous sodium bicarbonate (250 mL) to correct acidosis, intravenous propofol (0.5 g) for sedation, intravenous norepinephrine (16 mg) to stabilize blood pressure, intravenous methylprednisolone (40 mg) to control inflammation, intravenous amiodarone (150 mg) to regulate heart rate, and mechanical ventilation. During treatment, the patient experienced ventricular fibrillation, which was reversed using chest compressions, epinephrine, and defibrillation. Due to persistent circulatory instability, venoarterial extracorporeal membrane oxygenation (VA-ECMO) was initiated via the right femoral vessels under ultrasound guidance, and the patient was admitted to the ICU.

**TABLE 1:** Key laboratory test results of emergency department.

Category	Parameter	Result	Reference range	Clinical significance
Myocardial & cardiac function	Troponin I	15.400 ng/mL	<0.04 ng/mL	Markedly elevated, indicating acute myocardial injury
	NT-proBNP	300 ng/L	<125 ng/L	Reflects of heart failure
Acid-base & blood gas	Arterial lactate	14.50 mmol/L	0.5–1.6 mmol/L	Severe lactic acidosis, indicating tissue hypoperfusion
	pH	7.14	7.35–7.45	Severe acidosis
	HCO_3_^–^	15 mmol/L	21–25 mmol/L	Metabolic acidosis
Electrolytes	Potassium (K^+^)	2.80 mmol/L	3.5–5.0 mmol/L	Hypokalemia
	Sodium (Na^+^)	140 mmol/L	135–145 mmol/L	Normal
	Ionized calcium (Ca^2 +^)	1.0 mmol/L	1.15–1.29 mmol/L	Affects myocardial contractility and electrical activity
Renal/liver function & inflammation	Creatinine (Cr)	110 μmol/L	46–92 μmol/L	Assessment of renal function
	Aspartate aminotransferase (AST)	27 U/L	0–35 U/L	Normal
	C-reactive protein (CRP)	15.1 mg/L	<5.0 mg/L	Elevated inflammatory marker
	Procalcitonin (PCT)	4.84 ng/mL	<0.05 ng/mL	Aids in discriminating infectious etiology
Coagulation profile	D-dimer	17.15 μg/mL	<0.5 μg/mL	Markedly elevated, suggesting coagulation activation/fibrinolysis
	Platelet count (PLT)	202 × 10^9^/L	125–350 × 10^9^/L	Normal
	Prothrombin time (PT)	11.6 s	9.2–13.9 s	Normal
Others	Blood glucose (Glu)	32 mmol/L	3.9–6.1 mmol/L	Stress hyperglycemia is common

**FIGURE 1:**
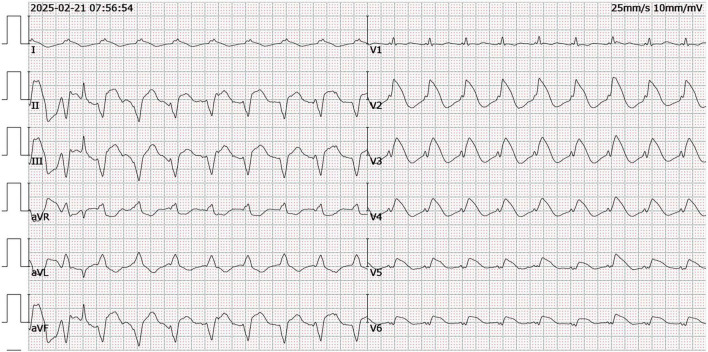
Electrocardiogram. The electrocardiogram indicates that the patient is experiencing ventricular tachycardia.

Given the marked elevation of troponin and the occurrence of ventricular fibrillation, acute myocardial infarction was initially suspected. However, emergency coronary angiography performed that night revealed no significant coronary stenosis. As this patient was a young woman with no prior medical history and had recently experienced acute urticaria, it is hypothesized that a viral infection might have caused the urticaria and concurrently led to fulminant myocarditis. Therefore, on the day of admission, she received 80 mg of methylprednisolone IV every 8 h, continuous renal replacement therapy (CRRT) for pulmonary edema, and daily intravenous immunoglobulin (10 g). Additionally, she was administered 4.5 g of piperacillin-tazobactam every 8 h, esmolol (800 mg via micro-pump) to control heart rate, and esomeprazole (40 mg every 12 h) for gastric protection. Despite this regimen, troponin levels continued to rise, reaching 60.057 μg/L, and recurrent ventricular fibrillation occurred. The patient’s condition progressively worsened, prompting further investigations.

Given the acute hemodynamic collapse, severe acidosis, and hypermetabolic state, other endocrine emergencies, including thyroid storm and adrenal cortical crisis, were considered in the differential diagnosis. However, the absence of fever or significant thyroid function abnormalities ruled out thyroid storm, and normal cortisol levels with low potassium levels were inconsistent with adrenal cortical crisis. On the seventh day after admission, abdominal CT revealed a 32 × 37 mm left adrenal mass, raising suspicion for a pheochromocytoma crisis (Figure 2A). Methylprednisolone was immediately discontinued. After discussions with the family, the patient was transferred to a higher-level hospital for further management. Upon transfer, the administration of norepinephrine was halted, and blood tests conducted 24 h later revealed elevated levels of metanephrines (451.10 pg/ml, normal range < 62 pg/ml) and normetanephrine (1091.40 pg/ml, normal range < 145 pg/ml). This confirmed the diagnosis of pheochromocytoma. Despite ongoing VA-ECMO support, an intensified preoperative pharmacological regimen was initiated to achieve adrenergic blockade. This included intravenous phentolamine (20 mg via syringe pump) for immediate alpha-blockade, supplemented with oral phenoxybenzamine (10 mg twice daily) for sustained effect, along with volume expansion. Once adequate alpha-blockade was achieved, beta-blockade was introduced to manage tachycardia and ventricular arrhythmias using esmolol (1000 mg via syringe pump) and oral propranolol (10 mg three times daily). All medications were titrated under continuous invasive hemodynamic monitoring. After 10 days of optimized medical management and stabilization, the patient underwent laparoscopic resection of the left adrenal mass under ongoing ECMO support (Figure 2B). Pathology confirmed the diagnosis of pheochromocytoma, with histopathological results as follows: CKpan-, Ki-67(+; 3%), Melan A(+), CgA(+), Syn(+), S-100 (localized +), SDHB(+). Postoperatively, ECMO was removed on postoperative day 10, and the tracheal tube was extubated on day 15. After approximately 25 days of treatment, the patient was discharged in stable condition. The entire treatment process is summarized in Figure 3. Following discharge, the patient was enrolled in a structured postoperative follow-up program. Her compliance with follow-up and tolerance were both good. No adverse events occurred. Nearly 1 year after surgery, her clinical condition has significantly improved. Oncologically, plasma metanephrine levels have normalized, and no tumor recurrence has been observed. Cardiovascularly, her blood pressure remains stable without the need for antihypertensive medications. Serum troponin and B-type natriuretic peptide levels have returned to normal, and serial echocardiograms confirm complete recovery of left ventricular systolic function. Looking back on her such thrilling experience, she expressed her gratitude for the positive treatment she received. These findings highlight the considerable reversibility of pathophysiological changes, even in severe cases of cardiogenic shock and myocardial injury leading to cardiac arrest, following the removal of the causative tumor. This case emphasizes the necessity of long-term, comprehensive follow-up, not only to confirm cure but also to fully assess the long-term prognosis after a critical illness. The patient was informed of the rarity and educational value of her case, and she consented to the publication of her anonymized clinical details, imaging, and outcomes for the advancement of medical knowledge.

**FIGURE 2:**
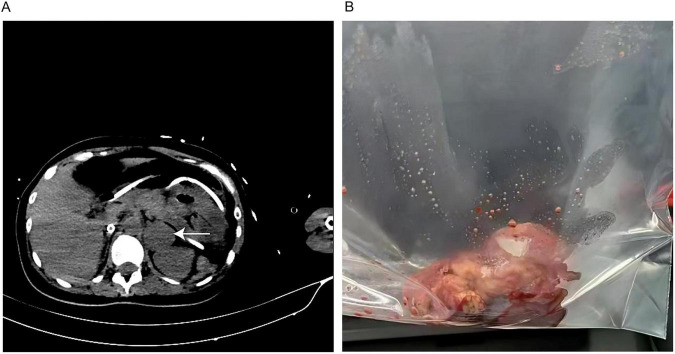
Adrenal pheochromocytoma. **(A)** The abdominal CT scan indicated a mass in the left adrenal gland (32 × 37 mm). **(B)** The specimen of the pheochromocytoma removed during the operation.

**FIGURE 3:**
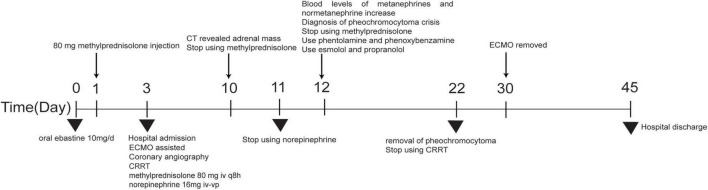
Patient hospitalization timeline.

## 3 Discussion

This case report details a pheochromocytoma crisis in a patient who developed a pheochromocytoma crisis and cardiac arrest following intravenous methylprednisolone administration. Laboratory investigations revealed significant lactic acidosis and elevated troponin levels. Coronary angiography and blood microbial RNA analysis showed no abnormalities. To rule out other potential pathogenic microorganisms, pathogen metagenomic sequencing (DNA + RNA), also known as mNGS, was utilized. This approach leverages next-generation sequencing technology to perform shotgun sequencing of DNA or RNA from clinical samples, allowing for unbiased detection of a wide range of pathogens, including viruses, bacteria, fungi, and parasites. However, the mNGS results in this case showed no significant abnormalities. Despite anti-inflammatory and anti-infective treatments, along with ECMO for circulatory support, the patient’s troponin levels continued to rise, and recurrent ventricular fibrillation occurred. These developments prompted an abdominal CT scan, which identified an adrenal mass, strongly suggesting a pheochromocytoma crisis. Methylprednisolone was immediately discontinued, and blood levels of metanephrines and normetanephrines were measured, leading to the diagnosis of pheochromocytoma (1). Following laparoscopic adrenalectomy, the patient gradually recovered and was discharged.

A pheochromocytoma crisis is characterized by the uncontrolled and excessive secretion of catecholamines by the tumor, leading to severe hypertension, organ dysfunction, and potentially fatal outcomes (6). Commonly recognized triggers include anesthesia, surgical procedures, direct tumor manipulation, and stressful events (9). This case report describes a pheochromocytoma crisis induced by methylprednisolone injection, consistent with several previously documented cases. David et al. (8) reported a pheochromocytoma crisis with severe lactic acidosis induced by dexamethasone. After β-adrenergic receptor blockade, the tumor was successfully removed, indicating that steroid use can expedite the onset of pheochromocytoma crises. Alejandro et al. (10) described four cases of glucocorticoid-induced pheochromocytoma crises, suggesting that exogenous glucocorticoids may unpredictably trigger these events. Experimental models have clarified the mechanisms by which glucocorticoids provoke catecholamine crises in pheochromocytomas, offering valuable translational insights. Goodman et al. (11) conducted *in vitro* studies using rat pheochromocytoma (G1) cell lines, revealing that glucocorticoids upregulate tyrosine hydroxylase activity via glucocorticoid receptors, thereby enhancing catecholamine synthesis. Similarly, organotypic brainstem cultures showed that corticosterone increased dopamine β-hydroxylase expression, promoting dopamine conversion to norepinephrine (12). Ex vivo and animal models further demonstrated that glucocorticoids increase α_1_ and β-adrenergic receptor density and affinity in vascular smooth muscle and endothelial cells, amplifying post-receptor signaling and enhancing vascular responsiveness to catecholamines (13). Additionally, glucocorticoids inhibit catechol-O-methyltransferase (COMT), slowing catecholamine degradation and prolonging their hemodynamic effects, both *in vivo* and *in vitro* (14). These mechanisms collectively explain how exogenous glucocorticoids, such as methylprednisolone in this case, can induce a substantial catecholamine surge in susceptible individuals, linking experimental findings to clinical vigilance.

Notably, pheochromocytomas often cause severe hypertension (15). However, in this case, the patient’s blood pressure did not significantly increase. During ECMO support, blood pressure remained within 79-142/72-109 mmHg, without the severe hypertension typically seen in pheochromocytoma crises. The reason for this atypical presentation is uncertain, but several hypothetical factors could be considered, albeit with caution as they remain unproven. First, VA-ECMO provides non-pulsatile flow that might theoretically dampen the hemodynamic impact of catecholamine-induced vasospasm on central arterial pressure (16). Second, prolonged cardiac arrest could potentially lead to transient adrenal medullary ischemia or functional exhaustion, possibly attenuating acute catecholamine release (17). In addition, CRRT, used for pulmonary edema, might have contributed to lowering circulating catecholamine levels. We emphasize that these are speculative possibilities rather than established mechanisms, and further studies are needed to explore the hemodynamic effects of mechanical support in pheochromocytoma crisis. Nonetheless, these considerations may help clinicians appreciate the complex and sometimes atypical presentation of this condition.

An important educational lesson from this case relates to the use of β-blockers before establishing α-blockade. β-adrenergic receptor antagonists should never be administered to patients with suspected or confirmed pheochromocytoma without prior or concurrent adequate α-adrenergic blockade, due to the risk of unopposed α-mediated vasoconstriction, which can precipitate hypertensive crisis, pulmonary edema, or cardiogenic shock. However, in the real-world emergency setting-particularly when pheochromocytoma is not initially suspected-β-blockers may be given empirically for tachyarrhythmias. In our patient, esmolol was administered under the working diagnosis of fulminant myocarditis. In retrospect, the subsequent hemodynamic instability and refractory ventricular arrhythmias may have been exacerbated by this unopposed β-blockade. This experience highlights two critical points: First, pheochromocytoma should be considered in the differential diagnosis of any patient presenting with unexplained cardiogenic shock, severe lactic acidosis, or refractory ventricular arrhythmias, even without prior hypertension. Second, when the diagnosis remains uncertain, if β-blockade is deemed absolutely necessary for life-threatening arrhythmias, it should be used with extreme caution-preferring an ultra-short-acting agent (e.g., esmolol) and only after ensuring that α-blockade is already in place or immediately available. Alternatively, other rate-controlling agents (e.g., amiodarone, magnesium, or lidocaine) might be considered as temporizing measures while diagnostic evaluation proceeds.

It is important to acknowledge that the causal role of methylprednisolone in this patient’s crisis, while plausible, cannot be definitively established. Several alternative or contributing triggers deserve consideration. First, the acute urticaria and the associated systemic inflammatory response may have acted as physiological stressors capable of provoking tumor activity. Second, the emergent medical procedures-including endotracheal intubation, mechanical ventilation, and central line placement-are well-known stressors that can precipitate a pheochromocytoma crisis. Third, the patient received multiple medications concurrently (e.g., norepinephrine and amiodarone), some of which might have influenced catecholamine dynamics or hemodynamic responses. Therefore, while methylprednisolone remains a likely contributing factor based on temporal association and known biological mechanisms, the crisis should be viewed as potentially multifactorial in origin. A more cautious interpretation is warranted, avoiding overstatement of a direct causal link.

Postoperative histopathological and immunohistochemical analysis in this case revealed the following markers: CKpan-, Ki-67(+; 3%), Melan A(+), CgA(+), Syn(+), S-100 (localized +), and SDHB(+). The positivity for CgA, Syn, and Melan A supports neuroendocrine differentiation, while localized positivity for S-100 indicates the presence of supportive cells with an uneven and discontinuous distribution, suggesting mild atypical features. The Ki-67 proliferation index was 3%, which is relatively low and generally considered to be associated with more indolent tumor behavior; however, it is important to note that a low Ki-67 index alone does not reliably exclude the possibility of metastatic potential. Similarly, while positive SDHB immunohistochemical staining suggests a reduced likelihood of an underlying SDHx-related genetic syndrome (which predisposes to higher risks of malignancy and multifocal disease), it does not completely rule out other hereditary susceptibility or the future development of metastases. Predicting the metastatic risk of pheochromocytoma remains challenging, and no single immunohistochemical marker or combination thereof can definitively determine biological behavior. Given these limitations, and to more comprehensively assess the patient’s long-term prognosis, referral for genetic counseling and testing (including germline testing for SDHx, RET, VHL, NF1, and MAX genes) is warranted. Furthermore, irrespective of the favorable histopathological features, lifelong annual clinical and biochemical follow-up is recommended for all patients with pheochromocytoma to monitor for potential recurrence or metastasis. The patient in this case has been enrolled in such a surveillance program, with no evidence of disease recurrence nearly 1 year post-surgery.

This case report has certain limitations, mainly due to its retrospective nature and the emergent circumstances of the patient’s presentation. First, as the patient presented with a fulminant pheochromocytoma crisis and cardiac arrest, requiring immediate ECMO and intensive care, the diagnostic process was expedited and prioritized on life-saving interventions. Consequently, specialized functional tests, such as the clonidine suppression test or iodine-131 metaiodobenzylguanidine (^131^I-MIBG) scintigraphy, which are often used in the elective workup of pheochromocytomas, were not performed. Additionally, tests for urinary catecholamines or their metabolites were not conducted, which would have provided more accurate diagnostic data. Lastly, the fresh tumor specimens were not placed on a flat surface for photographic documentation or precise measurement of tumor weight, potentially affecting the accuracy of tumor presentation.

## 4 Conclusion

To our knowledge, this is one of the few reported cases in which a pheochromocytoma crisis with cardiac arrest occurred shortly after methylprednisolone injection, suggesting a possible triggering role. However, the multifactorial nature of such crises should be recognized, and the causal relationship should not be overstated. This case emphasizes the importance of considering pheochromocytoma in the differential diagnosis for any unexplained severe heart failure, refractory shock, or cardiac arrest, and highlights the need for caution when administering glucocorticoids to patients with suspected pheochromocytoma.

## Data Availability

The original contributions presented in the study are included in the article/supplementary material, further inquiries can be directed to the corresponding author.
